# The inflammatory cytokine tumor necrosis factor modulates the expression of *Salmonella *typhimurium effector proteins

**DOI:** 10.1186/1476-9255-7-42

**Published:** 2010-08-12

**Authors:** Jun Ma, Yong-guo Zhang, Yinglin Xia, Jun Sun

**Affiliations:** 1 Department of Medicine, Gastroenterology & Hepatology Division, University of Rochester, 601 Elmwood Avenue, Rochester, NY 14642, USA; 2 Department of Microbiology and Immunology, University of Rochester, 601 Elmwood Avenue, Rochester, NY 14642, USA; 3 Department of Biostatistics and Computational Biology, University of Rochester, 601 Elmwood Avenue, Rochester, NY 14642, USA; 4 James Wilmot Cancer Center, University of Rochester, 601 Elmwood Avenue, Rochester, NY 14642, USA

## Abstract

Tumor necrosis factor α (TNF-α)is a host inflammatory factor. Bacteria increase TNF-α expression in a variety of human diseases including infectious diseases, inflammatory bowel diseases, and cancer. It is unknown, however, how TNF-α directly modulates bacterial protein expression during intestinal infection and chronic inflammation. In the current study, we hypothesize that *Salmonella *typhimurium senses TNF-α and show that TNF-α treatment modulates *Salmonella *virulent proteins (called effectors), thus changing the host-bacterial interaction in intestinal epithelial cells. We investigated the expression of 23 *Salmonella *effectors after TNF-α exposure. We found that TNF-α treatment led to differential effector expression: effector SipA was increased by TNF-α treatment, whereas the expression levels of other effectors, including gogB and spvB, decreased in the presence of TNF-α. We verified the protein expression of *Salmonella *effectors AvrA and SipA by Western blots. Furthermore, we used intestinal epithelial cells as our experimental model to explore the response of human intestinal cells to TNF-α pretreated *Salmonella*. More bacterial invasion was found in host cells colonized with *Salmonella *strains pretreated with TNF-α compared to *Salmonella *without TNF-α treatment. TNF-α pretreated *Salmonella *induced higher proinflammatory JNK signalling responses compared to the *Salmonella *strains without TNF-α exposure. Exposure to TNF-α made *Salmonella *to induce more inflammatory cytokine IL-8 in intestinal epithelial cells. JNK inhibitor treatment was able to suppress the effects of TNF-pretreated-*Salmonella *in enhancing expressions of phosphorylated-JNK and c-jun and secretion of IL-8. Overall, our study provides new insights into *Salmonella*-host interactions in intestinal inflammation.

## Background

Tumor necrosis factor α(TNF-α)is a pleiotropic inflammatory cytokine with increased expression in many human diseases. These diseases include septic shock, cancer, AIDS, multiple sclerosis, diabetes, rheumatoid arthritis, and inflammatory bowel disease [1-6]. It is well documented that multiple factors from bacteria, viruses, and parasites stimulate production of TNF-α in the host [7-10]. Hence, in hosts with inflammatory diseases, enteric bacteria are potentially exposed to high levels of TNF-α.

Bacteria can sense signal molecules secreted by their hosts. This communication mechanism between bacterium is called "quorum sensing" (QS) [11,12]. QS utilizes hormone-like compounds referred to as autoinducers to regulate bacterial gene expression [13,14]. QS also applies to the communication between the host and bacteria [11]. However, it is unknown how TNF-α from host cells directly modulates bacterial protein expression during infection and chronic inflammation.

*Salmonella *is a leading cause of gastrointestinal disease worldwide. *Salmonella *uses the type three secretion system (TTSS), a needle-like protein transport device to inject virulence proteins into eukaryotic host cells. These virulence factors, called effectors, paralyze or reprogram the eukaryotic cell to the benefit of the pathogen [15-17]. The activity of TTSS effectors allows bacteria to invade non-phagocytic cells or inhibit phagocytosis, regulate pro-inflammatory responses, prevent autophagy, or modulate intracellular trafficking [18]. *Salmonella *effectors display a large repertoire of biochemical activities and modulate the function of crucial host regulatory molecules[19-22].

Effectors are encoded via specific pathogenicity island 1 (SPI-1) and 2 (SPI-2). Over 30 *Salmonella *effectors, including AvrA, SipA, SipB, Gog B, and SpVB, have been shown to manipulate a succession of key signaling transduction pathways and physiological functions of host cells [19]. AvrA, SipA, SipB, SopB, SopD, SopE, SopE2 are SPI-1 effectors. SipA, SipB, SopB, SopD, SopE, SopE2 and other effectors are known to induce membrane deformation and ruffling that triggers bacterial internalization, promoting invasion [19,23,24]. The SPI-2 effectors, such as Gog B and SpVB, promote bacterial replication and systemic spread [19-22]. Recent studies indicate that there may be interplay between SPI-1 and SPI-2 effectors [19]. Although *Salmonella *is one of the best characterized pathogens, it remains unknown how virulence effector gene expression changes in response to host factors, such as TNF-α.

In *Salmonella *strains, AvrA is an acid-inducible effector that is strongly correlated with food hygiene and food-borne infection [25-27]. Our publications and others' have demonstrated that AvrA is a multifunctional protein that plays a critical role in inhibiting inflammation, regulating epithelial apoptosis, and enhancing proliferation during bacterial infection [28-32]. Stimulation of inflammation by effectors is crucial for *Salmonella *to grow in the intestine [33]. Effectors, such as SipA, SopE, and SopB, are known to activate inflammation in host cells [24,34-41]. Un-controlled inflammation is harmful to the host, however, and eventually damages the niche occupied by Salmonella during infection. *Salmonella *secreted factor L (SseL) [42-44], SspH 1 [45], SptP, and AvrA may reverse the activation of signaling pathways induced by other *Salmonella *effectors [19,46,47].

Intestinal epithelial cells are physically linked by intercellular junctional complexes that regulate multiple functions including polarity, mechanical integrity, and signaling capacity [48]. *Salmonella *can invade and replicate within intestinal epithelial cells during the infection process [49]. Nontyphoidal *Salmonella *serotypes such as *Salmonella typhimurium *provoke an intense intestinal inflammatory response, consisting largely of neutrophil migration across the epithelial lining of the intestine [50,51]. Studies of *S. typhimurium*-infected laboratory animals and cultured epithelial cells have shown that bacteria rapidly enter epithelial cells after transient degeneration of the host cell surface microvilli and induce inflammatory responses [52-58]. Not surprisingly, the ability of *S. typhimurium *to enter epithelial cells constitutes a crucial step in pathogenesis. *Salmonella *invasion of the intestinal epithelium requires the virulence-associated TTSS [19,28,34,53,59]. Within the host intestine specialized antigen-sampling M cells, which reside in the epithelium overlying lymphoid tissues in the gut, are a preferred site of *Salmonella *invasion [60]. The factors involved in *Salmonella*-M cell interactions, however, are not well understood. Clearly, studying effectors can uncover important mechanisms of regulation in host-bacteria interaction.

A recent study demonstrated that *Salmonella *gastroenteritis increases short- and long-term risk of inflammatory bowel disease [61]. Chronic intestinal inflammation enhances TNF-α levels in the host [62]. Therefore, enteric *Salmonella *is potentially exposed to TNF-α. In the current study, we hypothesize that *Salmonella *senses the host inflammatory factor TNF-α and that TNF-α treatment modulates *Salmonella *TTSS effectors, thus changing the host-bacteria interaction. We investigated the gene expression of *Salmonella *effectors changed by TNF-α and responses of the human intestinal cells to TNF-α treated *Salmonella*. We verified the expression levels of some effector proteins by Western blots. Furthermore, we used human intestinal epithelial cells as our experimental model to explore bacterial invasion and the proinflammatory NF-κB and c-Jun N-terminal kinase (JNK) signaling pathways in response to *Salmonella *strains with or without TNF-α pre-treatment. We found that TNF-α treatment modulated effector expression in a differentiated manner. *Salmonella *strains pre-treated with TNF-α induced more bacteria internalization and a more severe inflammatory response in intestinal epithelial cells than untreated *Salmonella *strains. Our study provides new insights into host factor regulation of bacterial effector expression through inflammatory responses.

## Materials and methods

### Bacterial strains and growth conditions

*Salmonella *strains (listed in Table 1) include wild-type (WT), *S. typhimurium *ATCC 14028s, *S. typhimurium *PhoP^C ^[63], *Salmonella typhimurium *1344 (SL1344), and an AvrA mutant strain lacking the AvrA gene (SL1344AvrA-) (provided by Dr. Jorge Galan from Yale University) [25]. Wild-type *S. typhimurium *14028s AvrA - was generated in our laboratory based on previously published methods by Hamilton *et al*., and Miller *et al*. [64,65]. Briefly, the AvrA gene, flanked by upstream and downstream *Salmonella *chromosome sequences, was cloned into pMAK705 (chloramphenicol resistant). The construct plasmid was transformed into the *Salmonella *WT14028s strain by electroporation with a Gene Pulser apparatus (Bio-Rad, Munich, Germany) and grown at 30°C on chloramphenicol plates. Resulting colonies were then grown at 42°C to select for integrants. The integrants were subsequently grown at 30°C, the temperature at which the plasmid can leave the chromosome and autonomously replicate. AvrA gene deletion was screened by PCR. AvrA deletion was also verified by Western blot using the anti-AvrA antibody. The resulting strain was named SL14028s AvrA-.

**Table 1:** *Salmonella *strains used in this study

Name	Description	Reference or source
*Salmonella *SL14028s	Wild-type pathogenic *Salmonella typhimurium*	ATCC

SL14028s AvrA-	SL14028s without *AvrA*	Constructed in our lab

SL1344	Wild-type *Salmonella *SL1344 strain	Hardt et al.,1997

SL1344 AvrA-	SL 1344 mutation without AvrA gene	Hardt et al.,1997

PhoP^C^	Non-pathogenic complex regulator mutant derived from SL14028s	Miller et al., 1990

Bacteria were grown under the following conditions: non-agitated microaerophilicbacterial cultures were prepared by inoculation of 10 ml of Luria-Bertani broth with0.01 ml of a stationary phase culture with or without TNF-α (10 ng/ml), followed by overnight incubation(~18 h) at 37°C, as previously described [53]. Overnight cultures of bacteria were concentrated 33-fold in Hank's balanced salt solution (HBSS) supplemented with 10 mM HEPES, pH 7.4. The overnight cultures from the TNF-α pretreated *Salmonella *strains were washed thoroughly with HBSS 3 times to get rid of potential TNF-α residue in the media. The bacteria were then resuspended in fresh HBSS for cell lysis or colonization in the intestinal epithelial cells.

### Reverse transcription polymerase chain reaction (RT-PCR)

Total RNA was extracted from bacteria using a Qiagen RNeasy mini kit (Cat: 74104. Qiagen, Valencia, CA) according to the manufacturer's protocol. Total RNA was further digested with DNase I (Cat: 18068-015. Invitrogen, Carlsbad, CA, USA). RNA integrity was verified by gel electrophoresis. Extracted RNA yield and purity was then determined by measuring absorbance in the 220 nm to 350 nm range. From the resulting spectra, the concentration of nucleic acids was estimated using the absorbance values at 260 nm, while the purity of each sample was determined by calculating the 260/280 and 260/230 ratios. RNA reverse transcription was performedusing a SuperScript III kit (Invitrogen, Cat: 18080-051)according to the manufacturer's directions. cDNA reactionproducts were then used in a quantitative PCR reaction. The reaction mixture was subjected to 29 cycles of PCR amplification using Taq polymerase (Fermentas, Glen Burnie, Maryland. Cat: EP0404). All PCR primers (Table 2) were designedusing Lasergene software (DNAStar, Madison, WI). PCR products were separated on 2% agarose gels and densitometry readings of the DNA bands were taken using a Kodak IS2000R. The densitometry value of each PCR band was detected using KODAK MI 4.0.3. All expressionlevels were normalized to the bacterial reference gene, Mdh, of the same sample, using forward (5'-ATGAAAGTCGCAGTCCTCGGCGCTGCTGGCGG-3') and reverse (5'-ATATCTTTYTTCAGCGTATCCAGCAT-3')primers for malate dehydrogenase (Mdh) [66]. All PCR reactionswere performed in triplicate. The digital images are representative of the original data.

**Table 2:** PCR Primers for *Salmonella *effector proteins

Gene	Forward primers	Reverse primers	Access No.
*AvrA*	5'GAATGGAAGGCGTTGAATCTGC3'	5'TTGTGCGCCTTGAGTATGTTTGTAA3'	NP_461786.1

*gogB*	5' TTC ATA TTT CCC AGA TAG CTT AG 3'	5' TCT TGC CTT ACA TAA ACC ATA A 3'	NP_461519.1

*luxR*	5' GAA CTA TAT CGC TCC TCA TGA CA 3'	5' TCC CAA AGA ATA GGT GAG TGA TT 3'	YP_002265254.1

*luxS*	5' CAC ATC CGC CAT CGC CGC TTT C 3'	5' GTT TGC TGG CTT TAT GCG CGA CC 3'	YP_002227567.1

*pipB1*	5' AGA ATT GCA GCG GTT AAG TTT AC 3'	5' CTG GAG GAT GTC AAC GGG TGT 3'	NP_460061.1

*pipB2*	5' ACC TTC ACA ATC CGC CAT A 3'	5' TAC GAG TCA GTA AAG GCG ACC AT 3'	NP_461706.1

*sifA*	5' TAG GTA TGT GGG TAT GCG GTG GT 3'	5' CAA ATG ACG GCC ATG ATT AAG A 3'	NP_460194.1

*sifB*	5' CCC TGA GCG GTT ACA ACT C 3'	5' CGT CGT CAA TAG CTG TTA CAC CT 3'	NP_460561.1

*sipA*	5' TGT TCG GCT ATT ATC AAT CGT CT 3'	5' CGC AGC AAT CTT ACG CAC CT 3'	NP_461803.1

*sipB*	5' CTG ACT GGG CTG CGG TAT TCG TG 3'	5' CTG CGG TGG GAC TTG CGG TAA 3'	NP_461806.1

*sipC*	5' GCC TTC AGC ACC GAG TTT G 3'	5' ATG TCA CGA CTA AAG CGA ATG AG 3'	NP_461805.1

*slrP*	5' GAT ACG CAG AAT ACC CGA CAC CC 3'	5' CCG CCA TAA TCA GTT CCG CTA A 3'	NP_459778.1

*sopA*	5' ATT CAG ACA CGG CGA TGA TG 3'	5' TGG CGT CCG TCA GGT GAT AAG CA 3'	NP_461011.1

*sopB*	5' TGA GTA ACC CGA CGG ATA CCA GT 3'	5' AGC ATC AGA AGG CGT CTA ACC AC 3'	NP_460064.1

*sopD*	5' TTA CTA TCA AGA TGG ACG CTT CT 3'	5' GTG CAT TTC CCG TCA CTT 3'	NP_461866.1

*sopE2*	5' CGG CGT AAC CTC TTT CAT AAC GA 3'	5' AGG GTA GGG CGG TAT TAA CCA GT 3'	NP_460811.1

*sptP*	5' AGG CGT CTT CCA GCA TTC TAT TG 3'	5' GAT CAC CAG CCG TTA CCG TCT AC 3'	NP_461799.1

*spvB*	5' AAC TTA ATC CCT CCG CAA TAT CA 3'	5' CGT TCC CGC AAA GCT ACA 3'	NP_490529.1

*ssaB*	5' TTT AAA AGG CAT TCC ATT AAT TC 3'	5' TTT ATG GTG ATT GCG TAT TAC AT 3'	NP_460358.1

*ssaM*	5' ATG GAT TGG GAT CTC ATT ACT GA 3'	5' GGA ATA CCC TGG AAC GCT 3'	NP_460378.1

*sseF*	5' CGG CAA GTA ATA TAG TCG ATG GT 3'	5' AAG GGT GTT AGC GCA GTT AAG A 3'	NP_460369.1

*sseG*	5' CCG GAC TTG CGA AAC GAG TG 3'	5' CCC ATC CAT ACC GAA GCG AGT AA 3'	NP_460370.1

*sseI*	5' TCA TAT TGG AAG CGG ATG TC 3'	5' GGC CAT TCA GAT TAC TCA TAC CT 3'	NP_460026.1

*sseJ*	5' CAG GAA CAC GCC GAT AAG TTG A 3'	5' CCG CCA AAG TAT TGA CCA TAG GA 3'	NP_460590.1

*sseL*	5' GAA CGG GAT CAT CAG ATA TAG AC 3'	5' CCC AAT AGG ATA GTT TAC CGA 3'	NP_461229.1

*sspH2*	5' GGT GGG TCA GCG GGT TAC T 3'	5' CCT TTC ATA TTG GAA GCG GAT GT 3'	NP_461184.1

### Immunoblotting for bacterial SipA and AvrA

Bacteria were lysed in lysis buffer (50 mM Tris, pH 6.8, 100 mM dithiothreitol, 2% SDS, 0.1%bromophenol blue, 10% glycerol) and sonicated. Equal amounts of total proteins were loaded, separated by SDS-PAGE, and processed for immunoblotting with an anti-SipA antibody (generated by Dr. Ho-Young Kang, Pusan National University, Korea) or anti-AvrA antibody. For the anti-AvrA antibody, a 15-amino-acid peptide CGEEPFLPSDKADRY was designed based on AvrA amino acids 216-230. Two rabbits were injected with the peptide and a polyclonal antibody for AvrA was tested and purified, as previously described [30]. Immunoblotting was visualized by enhanced chemi-luminescence (ECL). Chemi-luminescent signals were collected and scanned from ECL Hyperfilm (Amersham Pharmacia Biotech) with a Scanjet 7400c backlit flatbed scanner (Hewlett-Packard Co., Palo Alto, CA). Bands were quantified using Kodak MI software (v.4.0.3). The digital images are representative of the original data.

### Intestinal epithelial cell culture

Human colonic epithelial HCT116 cells (American Type Culture Collection, Manassas, VA) were grown in DMEM (high glucose, 4.5 g/L) supplemented with 10% (vol/vol) fetal bovine serum, 50 μg/ml streptomycin, and 50 U/ml penicillin.

### *S. typhimurium *invasion of human epithelial monolayers

Infection of HCT116 cells was performed by a previously described method [53]. Bacterial solution (~20 bacteria/epithelial cell) was added and bacterial invasion was assessed after 1 hour. Cell-associated bacteria, representing bacteria adhered to and/or internalized into the monolayers, were released by incubation with 100 μl of 1% Triton X-100 (Sigma). Internalized bacteria were those obtained from lysis of the epithelial cells with 1% Triton X-100, 20 min after the addition of gentamicin (50 μg/ml). Gentamicin, an aminoglycoside antibiotic, does not permeate eukaryotic plasma membranes and is therefore cytolytic only to extracellular populations of bacteria while intracellular bacteria populations remain viable [67]. For both cell associated and internalized bacteria, 0.9 ml LB broth was then added and each sample was vigorously mixed and quantitated by plating for CFU on MacConkey agar medium.

### Immunoblotting for epithelial cell signaling

Intestinal epithelial cells were incubated with equal numbers of the indicated *S. typhimurium *strain (about 20 bacteria per epithelial cell) for 30 minutes, washed, and incubated in fresh DMEM for 30 minutes as previously described [53,68,69]. Cells were rinsed twice in ice-cold HBSS, lysed in protein lysis buffer (50 mM Tris, pH 6.8, 100 mM dithiothreitol, 2% SDS, 0.1%bromophenol blue, 10% glycerol), and sonicated. Equal amounts of protein were separated by SDS-polyacrylamide gel electrophoresis, transferred to nitrocellulose, and immunoblotted with one of the following primary antibodies: anti-p65 (Santa Cruz Biotechnology Inc., Santa Cruz, CA, USA), anti-IκBα, anti-JNK, anti-phospho-IκBα, anti-phospho-c-JUN (Cell Signal, Beverly, MA), or anti-β-actin (Sigma-Aldrich, Milwaukee, WI, USA) antibodies and visualized by ECL.

### Real-time quantitative PCR analysis of the IL-8 mRNA

Total RNA was extracted from epithelial cell monolayers usingTRIzol reagent (Invitrogen, Carlsbad, CA). RNA integrity was verified by gel electrophoresis. RNA reverse transcription was doneusing the iScript cDNA synthesis kit (Bio-Rad, Hercules, CA)according to the manufacturer's directions. The RT cDNA reactionproducts were subjected to quantitative real-time PCR usingthe MyiQ single-color real-time PCR detection system (Bio-Rad)and iQ SYBR green supermix (Bio-Rad) according to the manufacturer'sdirections. IL-8 cDNA was amplified by using primers to thehuman IL-8 gene that are complementary to regions in exon 1(5'-TGCATAAAGACATACTCCAAACCT) and overlapping the splice sitebetween exons 3 and 4 (5'-AATTCTCAGCCCTCTTCAAAAA). All expressionlevels were normalized to the GAPDH levels of the same sample, using forward (5-CTTCACCACCATGGAGAAGGC) and reverse (5'-GGCATGGACTGTGGTCATGAG)primers for GAPDH. Percent expression was calculated as theratio of the normalized value of each sample to that of thecorresponding untreated control cells. All real-time PCR reactionswere performed in triplicate. All PCR primers were designedusing Lasergene software (DNAStar, Madison, WI).

### *Salmonella*-induced human IL-8 secretion

HCT116 cells were cultured in DMEM, followed by incubation in *Salmonella*-containing HBSS (1.6 × 10^10 ^bacteria/ml) for 30 min, washed 3 times in HBSS, and incubated at 37°C for 6 hours. Cell supernatants were removed and assayed for IL-8 by ELISA in 96-well plates as described previously [53].

### Treatment with JNK inhibitor SP600125

To determine whether the effects of TNF is required for JNK, cells were treated with a JNK inhibitor SP600125 (EMD Biosciences, San Diego, CA). SP600125 (50 μM) was added directly to the culture medium one hours before *Salmonella *treatment. For Western blot assay, HCT116 (with SP600125 pretreatment) were incubated with *Salmonella *(SP600125 50 μM) 1 hour, washed three times in HBSS and incubated HBSS (SP600125 50 μM) for 1 hour, then harvested. Levels of indicated proteins were determined by Western blotting as described above. For *Salmonella *invasion and IL-8 ELISA: HCT116 (with SP600125 pretreatment) were incubated with *Salmonella *(SP600125 50 μM) 1 hour, washed three times in HBSS and incubated DMEM for 6 hours.

### Statistical analysis

Data are expressed as means ± SD. All statistical tests were 2-sided. P values of less than .05 were considered to be statistically significant. Differences between two samples were analyzed by a Student's t-test. Statistical analyses were performed using SAS version 9.2 (SAS Institute, Inc., Cary, NC).

## Results

### The alteration of *Salmonella *effector gene expression after TNF-α treatment

**Figure 1:**
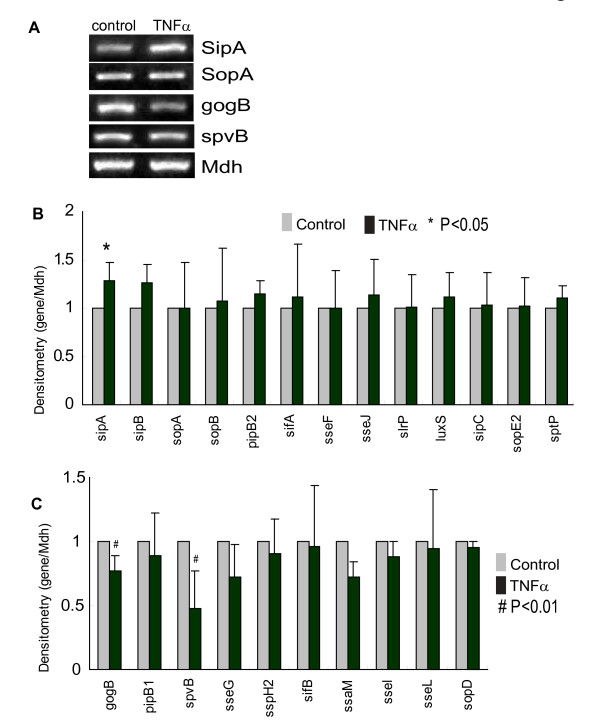
**Levels of effector gene expression determined by RT-PCR in *Salmonella *SL1344**. (A) Representative PCR results for effector mRNA expression. Mdh was used as the internal control. Control: *Salmonella *without treatment; TNF-α: *Salmonella *treated with TNF-α. (B) and (C) The relative intensity of PCR bands (Control vs. TNF-α-pretreated *Salmonella*). There were 3 repeated experiments performed in all controls and TNF-α treated groups. First, the densitometry value of each indicated effector gene was divided by the value for the Mdh band. Next, the obtained values were compared with the corresponding control values. The relative fold changes are shown in Figure 1B and 1C with the control group value set as "1" and compared to the TNF-α treated groups. Data are reported as the mean ± SD of three independent experiments. *P < 0.05 was considered significant.

We first tested whether TNF-α treatment changes the mRNA expression levels of *Salmonella *effectors. We used TNF-α at a concentration of 10 ng/ml, which is similar to the pathologic concentration in an inflamed intestine or patient serum [70]. Using RT-PCR, we investigated the mRNA expression of *Salmonella *effectors in the pathogenic *Salmonella *typhimurium SL1344 with or without TNF-α treatment. As shown in Fig. 1A, SipA was up-regulated by TNF-α, whereas gogB and spvB were down-regulated by TNF-α exposure (Fig. 1A). We tested 23 *Salmonella *effectors, those showing an upregulation of mRNA expression in response to TNF-αare shown in Fig. 1B and those that were downregulated following TNF-α exposure are shown in Fig. 1C. TNF-α significantly upregulated the mRNA expression of SipA, whereas it down-regulated the mRNA expression of gogB and spvB. Overall, this PCR data suggests that certain effectors are responsive to the host inflammatory factor TNF-α.

### Responses to TNF-α treatment in *Salmonella *strains with or without AvrA

**Figure 2:**
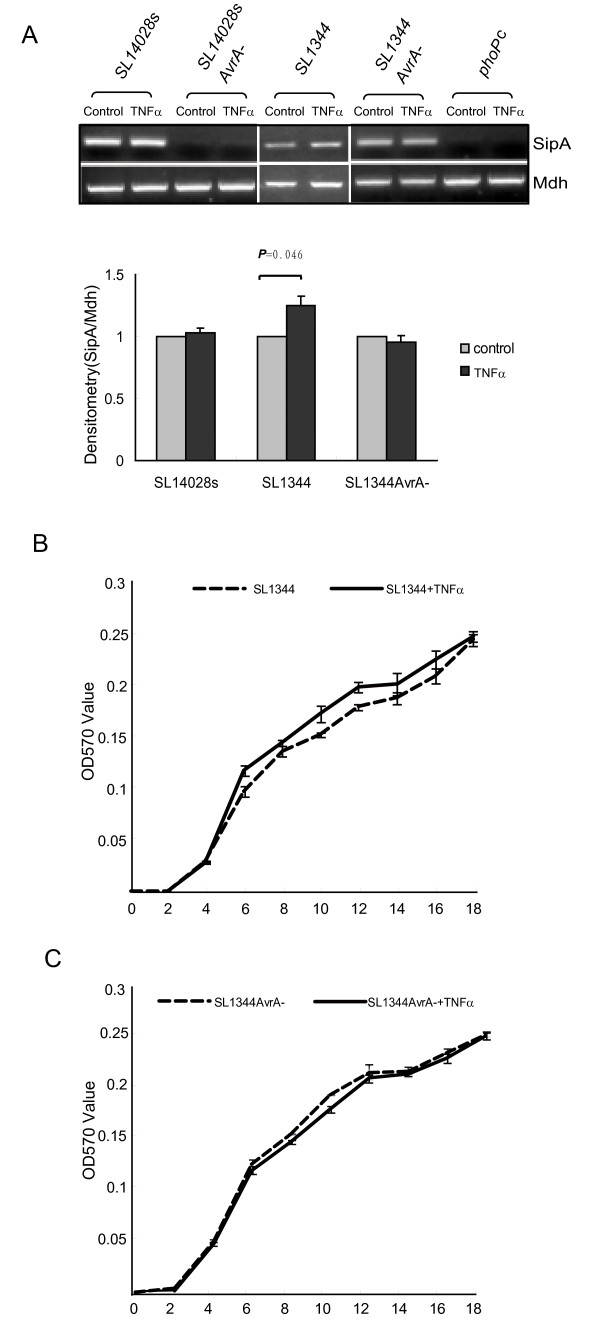
**The effects of AvrA deletion on effector expression**. SipA mRNA expression in the indicated *Salmonella *strains was determined by PCR. The relative intensity of the PCR bands was analyzed. Control: *Salmonella *without treatment; TNF-α: *Salmonella *treated with TNF-α. The data are reported as the mean ± SD of three independent experiments. *P < 0.05 was considered significant.

Our previous studies found that the *Salmonella *effector AvrA inhibits the proinflammatory NF-κB pathway (Collier-Hyams *et al*., 2002) and stabilizes β-catenin and IκBα [71]. We reasoned that the expression levels of AvrA in the bacterial strains may alter their responses to TNF-α treatment. Therefore, we tested effector expression levels in pathogenic *Salmonella *strains and corresponding AvrA mutants with or without TNF-α treatment. SL14028s with *AvrA *gene expression is known to express the AvrA protein only at low pH [27]. As shown in Fig. 2, SipA expression was not changed by TNF-α in SL14028s, whereas SipA mRNA in SL1344 was significantly elevated by TNF-α. PhoP^C ^is a mutation derived from SL1344 [63]. Interestingly, the SipA mRNA was undetectable in PhoP^C^.

To confirm that TNF-α pretreatment had no effects on bacterial growth, we measured the optical density (O.D.) of the bacteria in LB after TNF-α treatment for 18 hours. Over the still culture period, no significant difference was observed between the bacterial strain SL1344 with or without TNF-α pretreatment (Fig. 2B). Similar results were found in the SL1344 AvrA-strain with or without TNF-α treatment (Fig. 2C).

In Table 3, we summarize the changes of effector gene expression after TNF-α 18-hour treatment in *Salmonella *strains with or without AvrA expression.

**Table 3:** Bacteria effector gene expression after 18-hour treatment with TNF-α in *Salmonella *strains with or without AvrA

	SB1117 (AvrA-)	SB300 (with AvrA)	**phop**^**C**^	SL14028s (AvrA-)	SL14028s (with AvrA)
*SipA*	↓	↑*	ND	↑	↓

*SipB*	↑	↑	↓	↓	↑

*SipC*	↑	↑	ND	↓	ND

*sopA*	↓	↑	↓	↓	↓

*sopB*	↓	↓	↓	↑	↑

*sopD*	↑	↓	↓	↓	↓

*sopE2*	↑	↑	ND		↓

*sptP*	↑	↑	↓	ND	↑

*gogB*	↓	↓^#^	↓	↑	↓

*pipB1*	↑	↓	↑	↑	ND

*pipB2*	↑	↑	↑	↑	↓

*sifA*	↓	↓	↓	↑	↓

*sifB*	↓	↓	↓	↑	↓

*ssaM*	↑	↓	↓	↑	ND

*ssaB*	↓	ND	↓	↑	↓

*spvB*	↓	↓^#^	↑	ND	↓

*sseF*	↑	↑	↓	↑	↓

*sseG*	↑	↓	↓	↓	↑

*sseI*	↑	↓	↓	↑	↑

*sseJ*	↓	↑	↑	↑	↑

*sseL*	↑	↓	↓	↑	↓

*sspH2*	ND	↓	↓	↑	↓

*slrP*	↓	↑	↓	ND	↑

*luxS*	↓	↑	↑	↓	↑

* *P *< 0.05; ^# ^*P *< 0.01. ND: not detectable by PCR.

### Alteration of *Salmonella *effector proteins after TNF-α treatment

**Figure 3:**
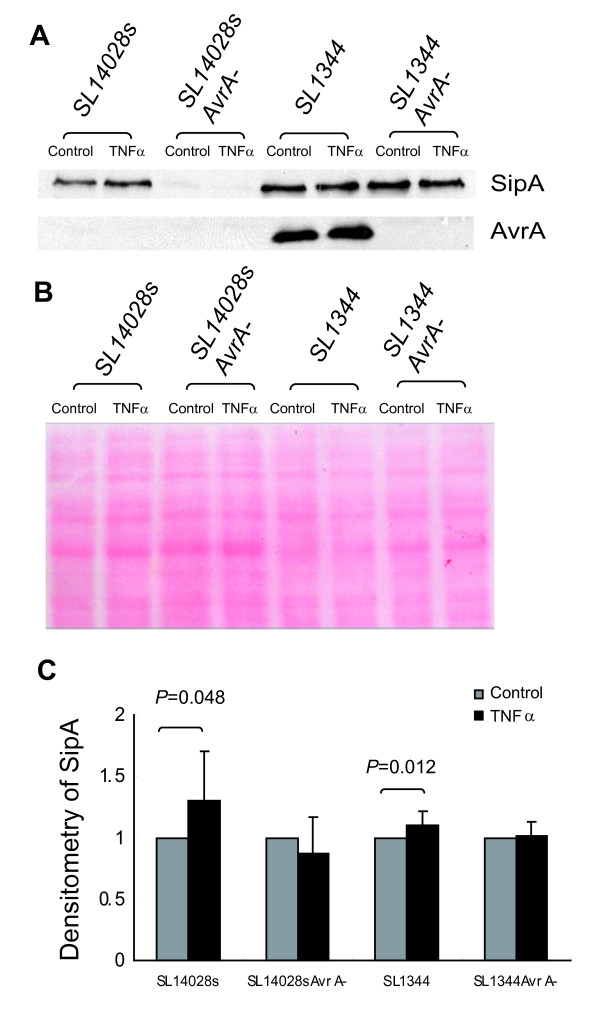
**SipA and AvrA protein expression**. (A) Western blot assay for the expression of SipA and AvrA. (B) Relative protein band intensity in Ponceau S Red staining. Data are reported as representative results from three independent experiments. (C) The relative intensity of the Western blot bands. The data are reported as the mean ± SD of three independent experiments. *P < 0.05 was considered significant.

Effector protein expression may be different from mRNA levels. We therefore examined strains of *Salmonella *to determine whether SipA protein levels respond to TNF-α treatment. As shown in Fig. 3, SipA expression was elevated by TNF-α in SL14028s and SL1344. To make sure the difference we observed was not due to protein loading variation, we stained the membrane with Ponceau S Red that indicates total protein levels (Fig. 3B). Relatively equal amounts of proteins in each lane were visible. We also found that SipA and AvrA could not be detected in the AvrA deletion strain derived from SL14028s (Fig. 3A). Without AvrA, SL1344 AvrA-did not alter SipA expression after TNF-α treatment. In addition, we generated an anti-AvrA antibody to detect the level of AvrA protein expression. SL14028s is known to express the AvrA protein only at low pH [26,27]. Therefore, we did not detect AvrA in the SL14028s group cultured in LB at pH 7.5. AvrA expression is high in the SL1344 strain and increased with TNF-α exposure. Taken together, we found that TNF-α significantly increased SipA protein expression in the pathogenic SL14028s and SL1344 strains (Fig. 3C).

### TNF-α pretreatment of *Salmonella *enhances invasion of host cells

**Figure 4:**
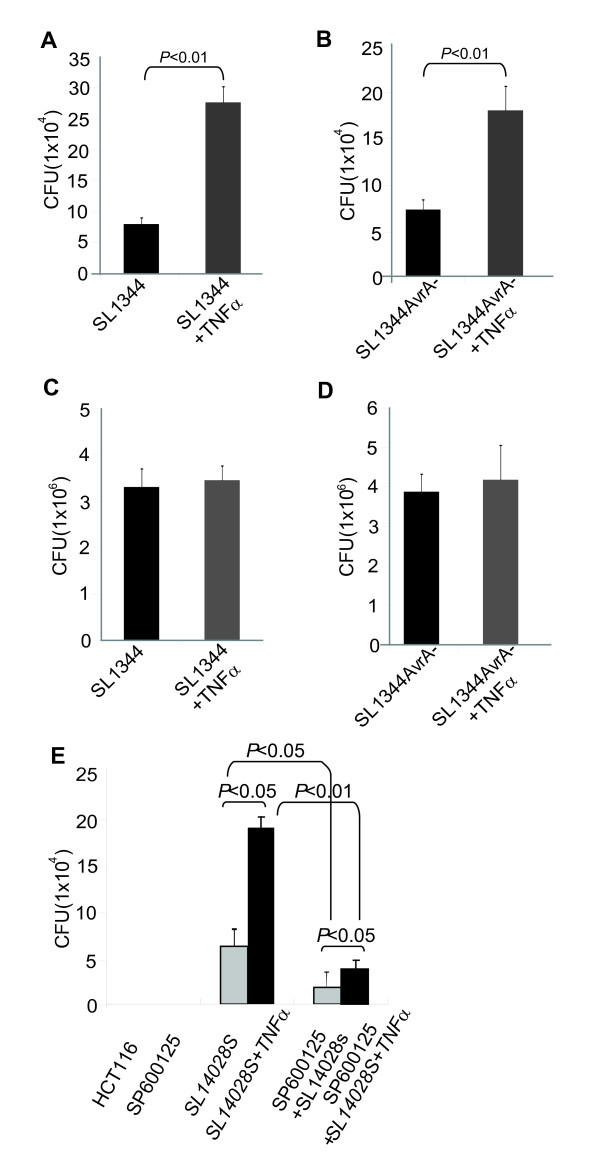
**TNF-α pretreatment of *Salmonella *contributes to enhanced bacterial invasion in human intestinal epithelial HCT116 cells**. (A) and (B) Number of internalized *Salmonella *(A: SL1344; B: SL1344 AvrA-) in the HCT116 cells. (C) and (D) Number of *Salmonella *associated with HCT116 cells. C: SL1344; D: SL1344 AvrA-. (E) Number of internalized *Salmonella *in the HCT116 cells with a JNK inhibitor SP600125 (50 μM) pretreatment. HCT116 cells were stimulated with *Salmonella *with or without TNF-α pretreatment for 30 min, washed, and incubated in fresh DMEM for 30 min. For both cell associated and internalized bacteria, 0.9 ml LB broth was then added and each sample was vigorously mixed and quantitated by plating for CFU on MacConkey agar medium. The mean ± SD is from three replicate experiments.

We then examined whether pre-treating *Salmonella *with TNF-α contributes to the physiological function of *Salmonella*, such as invasion. To determine whether TNF-α contributed to *Salmonella *invasion, we counted the number of *Salmonella *invading the human intestinal epithelial HCT116 cells. We found that TNF-α pretreatment of *Salmonella *increased the amount of internalized bacteria in epithelial cells versus untreated *Salmonella *SL1344 (Fig. 4A). In the *Salmonella *SL1344 AvrA-strain, we also found that TNF-α enhanced bacterial invasion of host cells (Fig.4B). Moreover, we examined the number of cell-associated bacteria, including bacteria adhered to and/or internalized into the epithelial monolayers. Our data showed no significant difference of *Salmonella *associated with epithelial cells with or without TNF-α pretreatment (Fig. 4C SL1344 and Fig. 4D SL1344 AvrA-). Furthermore, we used a JNK inhibitor, SP600125, to treat cells in order to confirm the enhanced bacterial invasion is related to the JNK pathway. Significantly less number of invaded bacteria was found in SL14028S group with SP600125 compared to the no-inhibitor groups (P < 0.05 Fig. 4E). However, invaded bacterial numbers in the TNF pretreatment group and non-TNF treatment group were still significantly different (P < 0.05 Fig. 4D), suggesting that SP600125 could not block the effect of TNF-pretreated *Salmonella *in enhancing invasion. These *in vitro *data indicates that TNF-α pretreatment changes the ability of *Salmonella *to internalize into host cells.

### TNF-α pretreated *Salmonella *changes the host response

**Figure 5:**
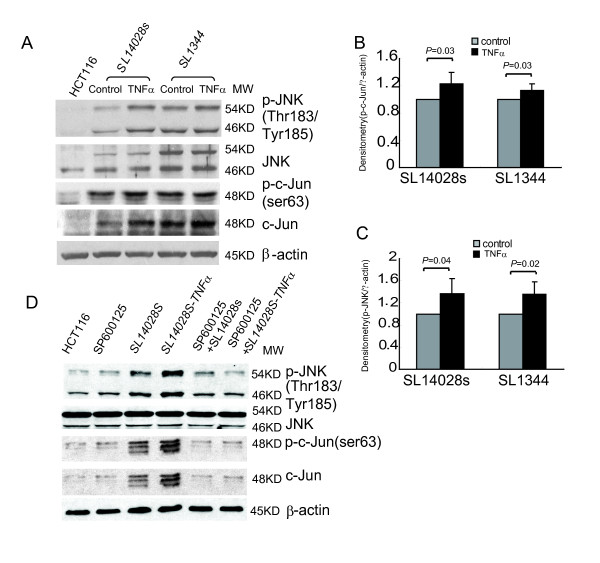
**JNK pathway is activated by *S. typhimurium *with or without TNF-α pretreatment**. A. The expression level of proteins associated with the JNK pathway in intestinal epithelial cells colonized with *Salmonella*. Intestinal epithelial cells were incubated with an equal number of the indicated *S. typhimurium *with or without TNF-α pretreatment. Epithelial cells were collected. Cell lysates were immunoblotted with the indicated antibodies. β-actin is an internal control for the Western blot. The data are reported as the mean ± SD of three independent experiments. (B) (C) Relative intensity of Western blot bands. *Salmonella *exposed to TNF-α induced higher activity of JNK pathway with enhanced p-JNK and p-c-Jun in the host cells. The data are reported as mean ± SD of three independent experiments. *P < 0.05 was considered significant. (D). The expression level of proteins associated with the JNK pathway in intestinal epithelial cells colonized with *Salmonella*. HCT116 cells were pretreated with a JNK inhibitor SP600125 (50 μM).

We further hypothesized that TNF-α treatment changes *Salmonella *effector protein expression, thus altering the host's inflammatory responses. The c-Jun N-terminal kinase (JNK) pathway is known to be regulated by the *Salmonella *effector AvrA [29,71]. *Salmonella *increases JNK phosphorylation [29]. We tested for the alteration of these two pathways as read-outs of inflammatory responses from host cells. We found that TNF-α pretreated *Salmonella *SL1344 could enhance c-JUN, p-c-JUN, and p-JNK expression in HCT116 cells (Fig. 5A). Statistical data further showed a significant difference in expression of p-c-JUN and p-JNK induced by *Salmonella *with or without TNF-α treatment (Fig. 5B and 5C). Moreover, we confirm the role of JNK pathway with a JNK inhibitor, SP600125. Inhibitor treatment blocked the enhancement of both p-c-JUN and p-JNK induced by *Salmonella *with or without TNF-α (Fig. 5D). In addition, we tested the activity of AP-1, a transcription factor which is a heterodimeric protein associated with c-Jun [72]. However, we did not find the difference in induction of AP-1 activity by *Salmonella *without TNF or with TNF-pretreatment (data not shown).

### IL-8 mRNA and protein levels in intestinal epithelial cells induced by *Salmonella *with or without TNF-α treatment

**Figure 6:**
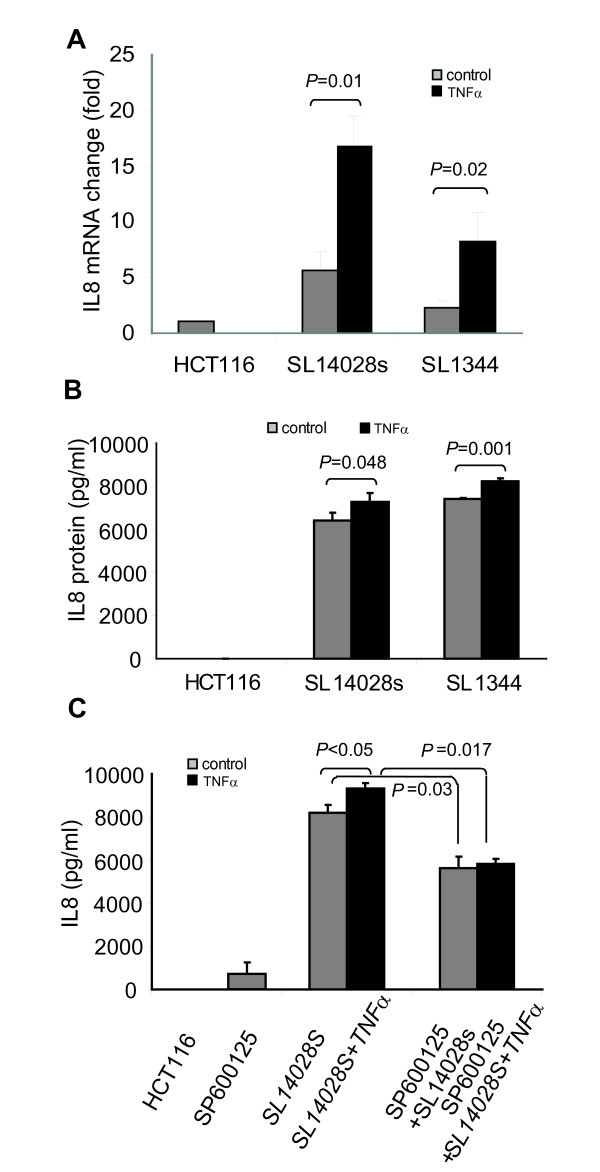
**TNF pretreatment of *Salmonella *contributes to enhanced IL-8 mRNA and proteins in human intestinal epithelial cells**. Cells were cultured in DMEM, followed by *Salmonella*-containing HBSS for 30 min, washed 3 times in HBSS, and incubated at 37°C for 6 hours. Total RNA was extracted for real-time PCR. Cell supernatants were removed and assayed for IL-8 by ELISA. (A) IL-8 mRNA levels in the HCT116 cells after colonization with TNF-pretreated *Salmonella*. (B) IL-8 protein secreted into the cell culture media in the HCT116 cells after *Salmonella *infection. (C) IL-8 protein secreted into the cell culture media in the HCT116 cells after *Salmonella *infection. HCT116 cells were pretreated with a JNK inhibitor SP600125 (50 μM). In a single experiment, samples were assayed in triplicate. The data are reported as mean ± SD of three independent experiments. *P < 0.05 was considered significant.

Cytokine IL-8 expression and secretion are common readouts for inflammatory responses in the host cells [73]. It is known that pathogenic *Salmonella *increases IL-8 through both transcriptional regulation and protein expression levels [58,71,73,74]. We reasoned that exposure to TNF-α makes pathogenic *Salmonella *more aggressive, inducing more severe inflammatory responses as compared to *Salmonella *without TNF-α treatment. We assessed the effect of TNF-α exposed *Salmonella *on IL-8 mRNA expression in human intestinal HCT116 cells. IL-8 mRNA real-time PCR showed that HCT116 cells significantly increased the level of IL-8 mRNA expression after TNF-α pretreated *Salmonella *colonization (Fig. 6A). In contrast, cells colonized with untreated *Salmonella *expressed less inflammatory IL-8 mRNA (Fig. 6A). Both pathogenic SL14028s and SL1344 had similar trends: TNF-α pretreated *Salmonella *induced significantly higher amounts of IL-8 mRNA, over 2.5 folds as compared to untreated *Salmonella *(Fig. 6A). Furthermore, we examined IL-8 protein secretion into the cell media caused by bacterial infection. As shown in Fig. 6B, an increase in IL-8 protein secretion was detected in the cell media after TNF-α pretreated *Salmonella *SL14028s colonization for 6 hours. In contrast, less IL-8 protein secretion was induced by untreated *Salmonella *SL14028s colonization (Fig. 6B). SL1344 had similar trends: TNF-α pretreatment induced significantly higher amounts of IL-8 secretion compared to untreated *Salmonella *(Fig. 6A). Overall, there is a significant difference of IL-8 secretion in cells colonized with *Salmonella *strains with or without TNF-α pretreatment. A possibility of the increased IL-8 could be due to the enhanced internalized bacteria after TNF pretreatment. We further tested the relationship between the bacterial loading, intercellular bacterial number and IL-8 secretion. However, we did not find that IL-8 secretion linearly related to the invaded bacterial numbers in the cells (data not shown). The enhanced bacterial invasion by TNF treatment and the increased IL-8 could be two different physiological effects in the host cells. Increased bacterial invasion is not necessary to induce increased IL-8 secretion.

To confirm the effect of TNF-pretreated *Salmonella *on IL-8 secretion is through the JNK pathway, we further used the inhibitor SP600125 to treat cells, significant less IL-8 was found in the SL14028S *Salmonella *with SP600125 group compared to the non-inhibitor group (Fig. 6C P < 0.03). SP600125 treatment was able to decrease the IL-8 secretion significantly in the SL14028 + TNF group v.s. the SP600125 + SL14028 + TNF group (Fig. 6C P = 0.017). There was significant difference in Sl14028S with or without TNF pretreatment (Fig. 6C P < 0.05). However, the difference between TNF pretreatment or no-TNF treatment was abolished after SP600125 pretreatment (Fig. 6C). Taken together, these IL-8 data indicate that TNF-α pretreated *Salmonella *stimulated more inflammatory responses in the intestinal epithelial cells through the JNK pathway.

## Discussion

The aim of this study was to determine the effect of TNF-α on *Salmonella *effector expression and the ability of TNF-α pretreated *Salmonella *to induce inflammatory responses in host epithelial cells. We investigated the regulation of *Salmonella *effectors in a variety of contexts, including mRNA expression, protein expression, and host-bacteria interaction Furthermore, we explored the response of human intestinal cells to TNF-α pretreated *Salmonella*. Bacterial invasion was enhanced in cells colonized with TNF-α pretreated *Salmonella*. *Salmonella *strains with TNF-α pretreatment induced higher proinflammatory responses compared to untreated *Salmonella*. Overall, our data show that TNF-α exposure makes *Salmonella *more virulent and enhances inflammation in host intestinal cells. This study provides a new insight into the *Salmonella*-host interaction in intestinal inflammation and infection.

Our study demonstrates that *Salmonella *senses the host inflammatory factor TNF-α and responds by changing its effector protein expression and enhancing its virulence, such as invasion. However, it is unknown how *Salmonella *senses TNF-α in the environment and whether *Salmonella *has a receptor for TNF-α. Recent findings have begun to reveal the molecular mechanisms by which bacteria can sense small innate immune molecules and modulate virulence gene expression. Wu *et al. *demonstrated that *Pseudomonas aeruginosa *recognizes host immune activation and responds by enhancing their virulence phenotype [75]. Also in *Pseudomonas*, Zaborina *et al*. showed dynorphin regulation of bacterial pathogenesis and cross-signaling between quorum sensing and quinolone signaling [76]. Norepinephrine modulates interactions between enterohemorrhagic *Escherichia coli *(EHEC) and the colonic epithelium by increasing bacterial adherence to the colonic mucosa [77]. EHEC uses a QS regulatory system to "sense" that it is within the intestine and then activates genes essential for intestinal colonization [13]. The QS system used by EHEC is known as the LuxS/autoinducer 2 (AI-2) system extensively involved in interspecies communication [13]. Given that eukaryotic cell-to-cell signaling typically occurs through hormones, and bacterial cell-to-cell signaling occurs through QS, QS may be used as a "language" by which bacteria and host cells communicate [13]. In *S. typhimurium*, the PhoQ sensor kinase is activated by host antimicrobial peptides. PhoQ then promotes the expression of virulence genes through a phosphorelay cascade [78]. However, it is still unknown how pathogenic *Salmonella *senses TNF-α, thus changing the expression of the bacterial effectors. Studies in *Pseudomonas *raise the possibility that TNF-α sensors or receptors for TNF-α are encoded for in the prokaryotic genome. Further studies on the *Salmonella *quorum sensing system and effector regulation will provide insights into this powerful and effective bacteria-host interaction.

Our data demonstrate that TNF-α exposure increases the expression of the *Salmonella *effector SipA. SipA contributes significantly to *Salmonella *host cell invasion *in vitro *and to *Salmonella *enterocolitis *in vivo *[35,41,79]. SipA also plays key role in maximizing pro-inflammatory responses [41]. Our bacterial invasion study further showed that cells colonized with TNF-α pretreated *Salmonella *had more internalized *Salmonella*. Moreover, *Salmonella *strains with TNF-α pretreatment induced higher proinflammatory responses, such as the activation of JNK and elevation of IL-8, compared to the *Salmonella *strains without TNF-α exposure. This observation is correlated with the enhanced expression of SipA in Salmonella exposed to TNF-α.

At the mRNA level SL1344 expressing AvrA has a significant ability to modify SipA in response to TNF-α, whereas SL14028s is less responsive (Fig.2A). We also found that an AvrA knockout strain derived from SL14028s had significantly decreased levels of SipA mRNA and protein. *Salmonella *SL14028s is known to be deficient in AvrA expression. AvrA protein expression was only detectable when SL14028s was cultured in low pH media [25-27]. The status of the effector AvrA may alter the expression of other effectors and the capacity of bacteria to induce host inflammation. Other factors in the environment may also contribute to the expression changes of *Salmonella *effectors. Although AvrA is known to regulate diverse bacteria-host interactions [28,29,71,80], the synergistic regulation of AvrA and other *Salmonella *effectors in response to the inflammatory status of the host cells remains unknown. Further investigations on the interaction of AvrA and its fellow effectors will help us to understand the network of *Salmonella *effectors in epithelial cell-bacteria cross-talk.

TNF-α exposure decreased the mRNA expression of SPI-2 effectors Gog B and SpVB, which are known to promote bacterial replication and systemic spread [19,20,22]. We did not examine the protein expression of these two proteins, while the cell culture system limited investigation into the physiologic relevance of the reduction of Gog B and SpVB. Long-term bacterial replication and systematic spread need to be examined in an *in vivo *model.

In summary, our current study answers the fundamental question of whether TNF-α expressed from host cells can change the expression level of *Salmonella *effectors, such as SipA, gogB, and spvB. *Salmonella *exposed to TNF-α induced more bacterial internalization, higher activity of JNK pathway with enhanced p-JNK and p-c-Jun in the host cells. As a consequence of the activation of the JNK pathway, the expression of inflammatory cytokines, such as IL-8, is higher in cells colonized with TNF-α pretreated *Salmonella*. Overall, *Salmonella *exposed to TNF-α caused enhanced inflammation in intestinal epithelial cells. We postulate that chronic inflammation with elevated TNF-α in host cells may change the behavior of pathogenic bacterial effectors and may make pathogens more virulent.

## Conclusions

We found that TNF-α treatment modulated effector expression in a differential manner. The expression of effector SipA was increased after TNF-α exposure in pathogenic *Salmonella*. Enhanced bacteria internalization and more severe inflammatory responses of intestinal epithelial cells were found after *Salmonella *strains were exposed to TNF-α. Activation of the JNK pathway significantly elevates and enhances inflammation in intestinal epithelial cells. As a consequence, the expression of inflammatory cytokines, such as IL-8, is high in cells colonized with TNF-α pretreated *Salmonella*. Our studies provide new insights into host factor TNF-α regulation of *Salmonella *effector expression in bacterial invasion and inflammatory responses.

## Competing interests

The authors declare that they have no competing interests.

## Authors' contributions

All authors read and approved the final manuscript.

JM: Participated in the experimental design, preparation of the RNA sample, PCR analysis, Western Blots, ELISA, acquisition of data, analysis and interpretation of data, and drafting of tables and figures. JM also helped to write Methods for the manuscript.

YZ: Participated in growing bacteria, bacterial invasion assays, Western Blots, ELISA, JNK inhibitor treatment, AvrA mutant strain establishment, interpretation of the data, statistical analysis, analysis and interpretation of data, and drafting of tables and figures.

YX: Performed statistical analysis and made critical revision of the manuscript for intellectual content.

JS: Participated in the study concept and design, acquisition of data, analysis and interpretation of data, material support, drafting and critical revision of the manuscript for important intellectual content. JS also obtained funding and supervised the study.
